# A scalable, chromatography-free, biocatalytic method to produce the xyloglucan heptasaccharide XXXG

**DOI:** 10.1186/s13068-024-02563-9

**Published:** 2024-08-20

**Authors:** Andrew M. Rodd, William M. Mawhinney, Harry Brumer

**Affiliations:** 1 Michael Smith Laboratories, University of British Columbia, 2185 East Mall, Vancouver, BC V6T 1Z4 Canada; 2 Department of Chemistry, University of British Columbia, 2036 Main Mall, Vancouver, BC V6T 1Z1 Canada; 3 BioProducts Institute, University of British Columbia, 2385 East Mall, BC V6T 1Z4 Vancouver, Canada

**Keywords:** Tamarind kernel powder, Carbohydrate-active enzymes, Xyloglucanase, Yeast, Galactose fermentation

## Abstract

Xyloglucan oligosaccharides (XyGOs) are highly branched, complex carbohydrates with a variety of chemical and biotechnological applications. Due to the regular repeating pattern of sidechain substitution of the xyloglucan backbone, well-defined XyGOs are readily accessed for analytical and preparative purposes by specific hydrolysis of the polysaccharide with *endo*-glucanases. To broaden the application potential of XyGOs, we present here an optimized, scalable method to access large quantities of galactosylated XyGOs by treatment of the bulk agricultural by-product, tamarind kernel powder (TKP), with a highly specific *endo*-xyloglucanase at high-solids content. Subsequent β-galactosidase treatment reduced XyGO complexity to produce exclusively the branched heptasaccharide XXXG (Xyl_3_Glc_4_: [α-D-Xyl*p*-(1 → 6)]-β-D-Glc*p*-(1 → 4)-[α-D-Xyl*p*-(1 → 6)]-β-D-Glc*p*-(1 → 4)-[α-D-Xyl*p*-(1 → 6)]-β-D-Glc*p*-(1 → 4)-D-Glc*p*). The challenge of removing the co-product galactose was overcome by fermentation with baker’s yeast, thereby avoiding chromatography and other fractionation steps to yield highly pure XXXG. This simplified approach employs many of the core concepts of green chemistry and engineering, enables facile production of 100 g quantities of XyGOs and XXXG for laboratory use, and serves as a guide to further production scale-up for applications, including as prebiotics, plant growth effectors and elicitors, and building blocks for glycoconjugate synthesis.

**Supplementary Information:**

The online version contains supplementary material available at 10.1186/s13068-024-02563-9.

## Background

The xyloglucans (XyG) comprise a family of complex polysaccharides found in the cell wall matrix of nearly all terrestrial plants [1, 2]. Xyloglucans also occur in large quantities as storage polysaccharides in the seeds of some species, e.g., tamarind and nasturtium [3]. All xyloglucans are built upon a β(1 → 4)-linked glucan backbone, which is highly substituted with α(1 → 6)-linked xylopyranosyl residues. Depending on the species of origin, two regular, repeating xylosylation patterns are commonly observed, XXXG-type and XXGG-type, where G represents an unbranched glucopyranosyl residue and X represents a branched [α-D-Xyl*p*-(1 → 6)]-β-D-Glc*p*-(1 → 4)- unit in the standard nomenclature [4, 5]. These core motifs are further elaborated in a species-dependent manner with galactosyl, fucosyl, arabinosyl, and acetate residues, resulting in a diversity of *ca.* 20 sidechain structures with unique single-letter identifiers [5–8].

**Fig. 1:**
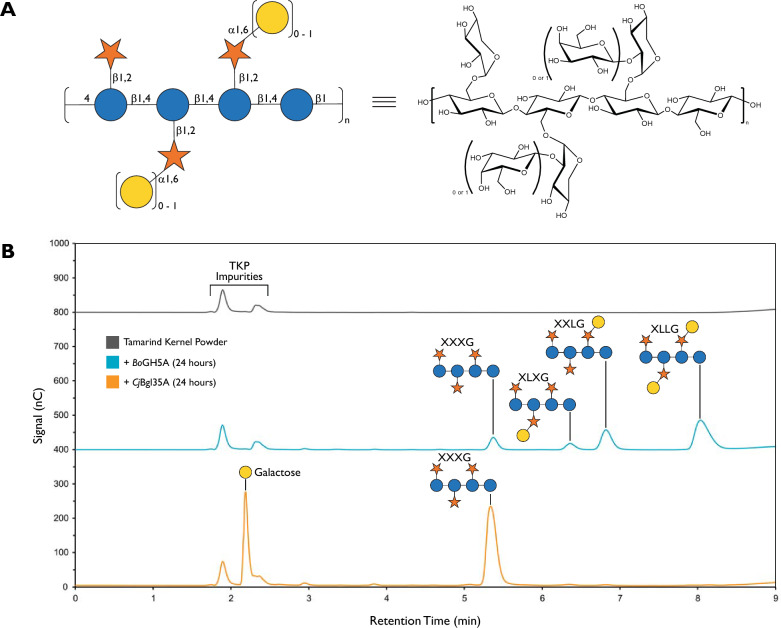
Structure of tamarind xyloglucan and enzymatic hydrolysis into oligosaccharides. **A** Structure of tamarind xyloglucan, including symbol nomenclature for glycans representation [48]. **B** HPAEC-PAD chromatograms of an example enzymatic hydrolysis of tamarind kernel powder (TKP) xyloglucan, using *endo*-xyloglucanase *Bo*GH5A (0.2 mg/g TKP) and β-galactosidase *Cj*Bgl35A (0.4 mg/g TKP) at room temperature (21–22 ºC)

Tamarind kernel powder (TKP), an agricultural byproduct from tamarind fruit processing, is a major industrial source of xyloglucan. Tamarind seed kernels contain *ca.* 50% w/w (galacto)xyloglucan [9], which is based on repeats of the oligosaccharides XXXG, XLXG, XXLG, and XLLG (where L represents a [β-D-Galp -(1 → 2)-α-D-Xylp -(1 → 6)]-β-D-Glcp -(1 → 4)- [Gal*p*(β 1–2)-Xyl*p*(α1-6)]-Glc*p* unit, Fig. 1) in the proportions *ca.* 1:0.5:2:3 [10, 11]. Crude TKP is used as animal fodder and as a textile sizing agent, while both TKP and purified tamarind xyloglucan have found applications in the food and pharmaceutical industries as rheology modifiers and gelling agents [12–14]. The strong interaction between tamarind xyloglucan and cellulose fibers has been used to improve paper properties, produce composite hydrogel materials and plant cell wall mimics, and attach chemical functional groups to cellulose surfaces [15–23].

Likewise, xyloglucan oligosaccharides (XyGOs), obtained by the controlled hydrolysis of XyG polysaccharide, are valuable for diverse applications. In unmodified form, XyGOs are useful substrates for enzymology [5] and have potential as plant growth effectors [24–26] and prebiotic food additives [27, 28]. XyGOs in free or protected forms are robust to chemical transformation, and therefore serve as valuable building blocks to produce structurally complex enzyme substrates (radiolabelled, fluorophoric, chromogenic, fluorogenic, and others) [29–38], covalent enzyme inhibitors [39, 40], and surfactants [11, 41–43]. Conveniently, many general *endo*-β(1→4)-glucanases (“cellulases”) and xyloglucan-specific *endo*-β(1→4)-glucanases (*endo*-xyloglucanases) hydrolyze tamarind XyG specifically at the unbranched glucosyl residue to produce a mixture of XXXG, XLXG, XXLG, and XLLG [44, 45]. This mixture can be separated by chromatography, including after per-*O*-acetylation, or further simplified by β-galactosidase treatment, for chemical synthesis and other applications [11, 32, 46, 47].

To date, the enzymatic production of XyGOs has generally been limited to laboratory scale, from dilute aqueous solution (10 g/L, *i.e.,* 1% w/w), due to the inherent solubility and rheological properties of XyG polysaccharide. A further challenge in the production of the pure heptasaccharide XXXG (Xyl_3_Glc_4_) is the need to remove the monosaccharide galactose following β-galactosidase hydrolysis. To facilitate the broader use of XyGOs in direct applications and chemical syntheses, we sought to develop a scalable, “green” biocatalytic process that reduces water inputs, avoids chemical and chromatographic steps, and reduces or eliminates the use of organic solvents [49, 50]. Here, we present a streamlined method to produce XyGOs and high-purity XXXG from crude TKP, which incorporates enzyme hydrolysis at high-solids content and galactose removal by fermentation with baker’s yeast as key steps. We applied this method on a 100 g scale in a laboratory setting, thereby demonstrating its potential for future facile scale-up toward kilogram amounts.

## Results and discussion

### Enzyme selection

XyGO production from TKP and other xyloglucans was first achieved using fungal *endo*-β(1→4)-glucanases, including both “cellulase” cocktails and monocomponent enzymes, and fungal β-galactosidases. Indeed, cellulase preparations from *Trichoderma* species and β-galactosidase preparations from *Aspergillus* species are commercially available from laboratory biochemical suppliers, and are thus attractive for routine use [11, 30, 32, 46]. Anecdotally, we have observed previously that these preparations can lead to unwanted degradation of the desired XyGOs, presumably due to contaminating activities in these crude preparations from native sources (Brumer Laboratory, unpublished observations). Hence, we compared the performance of the commercial fungal enzyme preparations with purified, recombinant bacterial glycosidases for the selective production of XXXG-type XyGOs. We chose the *Bacteroides ovatus* Glycoside Hydrolase Family 5 *endo*-xyloglucanase (*Bo*GH5A) and the *Cellvibrio japonicus* Glycoside Hydrolase Family 35 β-galactosidase (*Cj*Bgl35A), which are key enzymes in bacterial xyloglucan utilization systems with well-defined specificities and high activities [51, 52]. In all experiments, β-galactosidase treatment was performed after backbone hydrolysis because degalactosylation of XyG leads to aggregation and precipitation [13, 53, 54], which was anticipated to limit *endo*-(xylo) glucanase activity.

HPLC analysis indeed indicated that recombinant *Bo*GH5A produced the expected Glc_4_-based XyGOs, XXXG, XLXG, XXLG, and XLLG, as the exclusive products of crude TKP hydrolysis. On the other hand, the *Trichoderma* cellulase preparation yielded the same XyGOs as the major products, however peaks corresponding to both shorter and longer oligosaccharides were also observed (Figure S1). MALDI-MS was used to confirm these assignments of XXXG, XLXG/XXLG, XLLG ([M + Na]^+^ *m/z* calculated 1085.33, 1247.38, 1409.44, *m/z* observed 1084.8, 1247.0, 1409.0, respectively). Treatment of the XyGO mixture produced by *Bo*GH5A with *Cj*Bgl35A generated exclusively XXXG and galactose, due to hydrolysis of both t-Gal residues. β-galactosidase preparations from *Aspergillus oryzae* and *A. niger* were likewise effective at hydrolyzing t-Gal residues, but also caused extensive degradation of the desired XXXG product (Figure S1). MALDI-MS confirmed these observations as both digestion products contained XXXG ([M + Na]^+^ *m/z* calculated 1084.33, observed *m/z* 1084.8), while digestion with *A. oryzae* β-galactosidase contained extra peaks (observed *m/*z 790.2, 1379.0).

Correspondingly, SDS-PAGE analysis of the commercial enzyme preparations versus the recombinant enzymes showed multiple protein bands in the former (Figure S2). A key goal was to eliminate the requirement for additional oligosaccharide purification steps, including potentially tedious chromatography, toward a scalable method. Hence, recombinant *Bo*GH5A and *Cj*Bgl35A were chosen for subsequent optimization. Further activity and product yield measurements were not performed on the commercial enzymes, which were abandoned.

### TKP hydrolysis at high-solids content

The addition of water is necessary to solubilize XyG in TKP for enzyme hydrolysis, yet must ultimately be removed to produce a solid XyGO product. Purified XyG from TKP has a practical solubility limit for laboratory use of 1% w/v (10 mg/mL), whereas the solubility of Glc_4_-based XyGOs is at least tenfold higher. Therefore, we were interested to explore the direct hydrolysis of XyG from TKP in a slurry at high-solids content.

Initial range-finding experiments of substrate preparation and enzyme loading revealed that a 4% w/v slurry of TKP in water could be hydrolyzed to XXXG, XLXG, XXLG, and XLLG in 6 h at room temperature (21 ºC–22 ºC) using 20 µg/mL (0.2 mg enzyme/g TKP) *Bo*GH5A (data not shown, *cf.* Figure 1 and data for 10% TKP slurry below). Conveniently, performing the digestion at ambient temperature facilitated further scale-up, because it avoided heating the suspension to > 50 °C (typically used to dissolve purified tamarind XyG) and subsequent cooling prior to *endo*-xyloglucanase addition. We also observed that TKP slurries in water have a pH value of 6.4, similar to the pH optima of both *Bo*GH5A and *Cj*Bgl35A [51, 52], so additional buffering was not necessary, further simplifying the method. Following direct addition of *Cj*Bgl35A to 40 µg/mL (0.4 mg enzyme/g TKP), subsequent hydrolysis of galactosylated XyGOs, to produce exclusively the heptasaccharide XXXG, reached completion after an additional 6 h at room temperature.

With an interest to further reduce water input, we explored the hydrolysis of TKP at solid loadings of 4% to 20% w/v. At loadings higher than 20% w/v, the slurry was found to be too viscous for sufficient mixing by stirring with a magnetic stir bar, shaking, or end-over-end mixing. Quantitative analysis of XXXG by HPLC after sequential hydrolysis with *Bo*GH5A and *Cj*Bgl35A at constant enzyme loading (weight basis) showed that yields decreased from *ca.* 55% (w/w) for the 4% w/v TKP loading to *ca.* 43% at 20% w/v loading (Figure S3). The maximum theoretical yield is *ca.* 50–60% (w/w), based on reported amounts of XyG in TKP [55, 56]. At the highest solids content of 20% w/v TKP, the maximum theoretical concentration of XyGOs would be 100 g/L, so we hypothesized that the decrease in yield may have resulted from a combination of enzyme product inhibition and solubility limitations, including in the presence of insoluble residues from the crude TKP. In light of these results, we selected a TKP solids content of 10% as a compromise between water input and yield. As shown in Figure S4, these conditions resulted in complete hydrolysis to XXXG and galactose after 6 h of *Bo*GH5A treatment followed by 6 h of *Cj*Bgl35A treatment. For practical simplicity, subsequent experiments employed initial hydrolysis with *Bo*GH5A for 6 h, followed by addition of *Cj*Bgl35A and overnight incubation. No effort was made to inactivate or remove the *endo*-xyloglucanase during the degalactosylation step, because over-digestion of XXXG by *Bo*GH5A was not a concern (Figure S1).

### Galactose removal

Applications of XXXG, e.g., use as a prebiotic [27], plant growth enhancer [24–26], enzyme substrate [29, 30], or building block for glycoconjugate synthesis [11, 31–43], require the removal of galactose to isolate the pure oligosaccharide. Although this can be achieved practically on small scale by size exclusion chromatography [10, 24, 47], solvent, medium, and time requirements severely limit scalability. Hence, we investigated alternative methods for removing galactose following β-galactosidase treatment of XyGOs.

Selective precipitation of carbohydrates by addition of water-miscible organic solvents has been used previously to purify beet pectin after acid hydrolysis [57], and to separate different sizes of inulin and oligoglucosides [58]. Ethanol, in particular, is a relatively low-cost, low-toxicity solvent produced by microbial fermentation. Ethanol is also widely used to precipitate XyG for laboratory preparations, including purification from TKP and plant primary cell walls. Based on these precedents, we first explored the selective precipitation of XXXG with ethanol and acetone, taking advantage of the anticipated solubility difference of the heptasaccharide versus the monosaccharide galactose.

After removal of remaining solids by centrifugation from a digestion of 10% w/v TKP with *Bo*GH5A and *Cj*Bgl35A, addition of one portion of ethanol to the solution of XXXG (1:1 addition) failed to precipitate any significant amount of XXXG, nor galactose, as quantified by HPLC (Figure S5). Likewise, 5:1 addition of ethanol resulted in only minimal precipitation of XXXG (*ca.* 20%). At higher ratios of ethanol, i.e., 10:1 and 20:1, much more XXXG was precipitated (74% and 86%, respectively), although this precipitate also contained *ca.* 10% of the original amount of galactose (Figure S5). Thus, although this method is quite effective at separating XXXG from the majority of co-product galactose, it both fails to produce high-purity XXXG and leaves a significant amount of XXXG in solution, decreasing overall yield. Moreover, it requires a large total volume of ethanol to achieve significant XXXG precipitation (*e.g.,* 10–20 L ethanol for 1 of hydrolysate). As such, this is not ideal from the perspective of reducing solvent inputs and the corresponding equipment requirements for liquid handling. Precipitation using acetone yielded similar results using the same ratios (data not shown) and was therefore not explored further.

Inspired by recent work on the use of yeasts to remove galactose from galacto-oligosaccharide preparations by selective fermentation [59], we explored this as a potential route to produce pure XXXG. Indeed, it has been long known that *Saccharomyces cerevisiae* (“brewer’s yeast” or “baker’s yeast”) can ferment select monosaccharides including galactose into ethanol and carbon dioxide, but is unable to utilize many disaccharides and more complex oligosaccharides, including cellobiose (β-D-Glc*p*-(1 → 4)-D-Glc*p*) [60]. *S. cerevisiae* is also generally unable to ferment xylose [60]. In the absence of direct literature precedent, we reasoned that *S. cerevisiae* would be likewise unable to utilize the complex heptasaccharide XXXG. To establish the proof-of-concept with a readily available strain, we selected a popular consumer brand of baker’s yeast from a local grocery store. As a first step, we established that this strain was unable to degrade XyGOs produced from TKP by *Bo*GH5A, including an inability to remove pendant galactosyl residues (Figure S6).

**Fig. 2:**
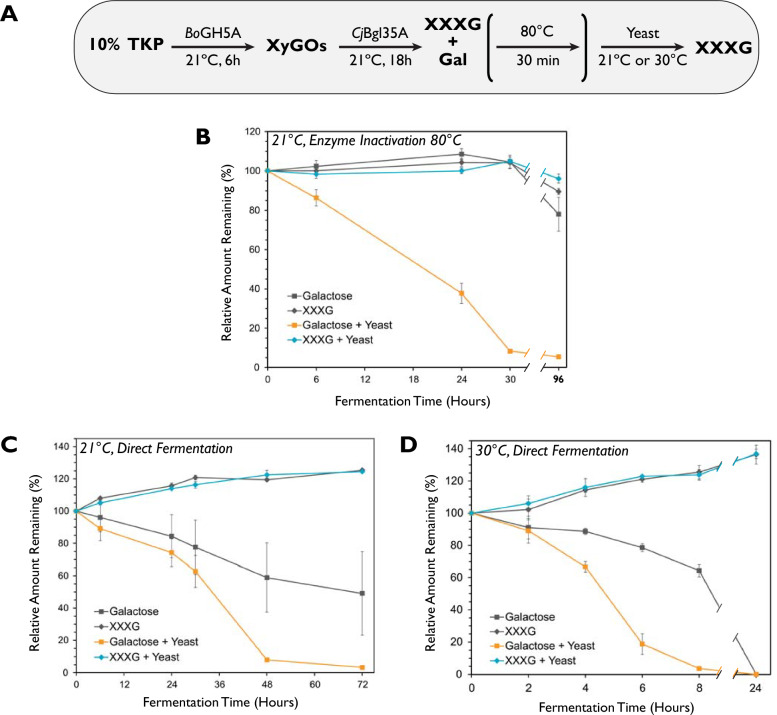
Removal of galactose from tamarind kernel powder (TKP) enzymatic digestions using fermentation by *Saccharomyces cerevisiae* (yeast). **A** Digestion scheme. **B**–**D** XXXG and galactose amounts quantified using HPAEC-PAD following **B** enzyme inactivation, addition of yeast, and incubation at 21 ºC, **C** addition of yeast without enzyme inactivation and incubation at 21 ºC, and **D** addition of yeast without enzyme inactivation and incubation at 30 ºC. In each case, grey lines represent control samples to which yeast was not added. *Bo*GH5A was loaded at 0.2 mg/g TKP and *Cj*Bgl35A at 0.4 mg/g TKP

Galactose metabolism by *S. cerevisiae* is induced and not constitutive [61], so we routinely primed cells in medium containing 2% w/v galactose, followed by briefly washing in phosphate-buffered saline to remove galactose and other media components before addition to TKP enzyme digests. In a first experiment, yeast was added to an overnight digest of 10% w/v TKP with *Bo*GH5A and *Cj*Bgl35A, after the enzymes had been inactivated by heat (80 ºC for 30 min, Fig. 2A). At the end of the digest, yeast cells were conveniently removed by the centrifugation step normally required to remove insoluble TKP residues. HPLC analysis revealed that the amount of galactose was reduced by over 90% after 30 h in samples to which the yeast was added, while the amount of XXXG remained unchanged (Fig. 2B). Importantly, these data confirmed that *S. cerevisiae* was able to remove galactose effectively from the mixture, yet was unable to break-down and metabolize the xyloglucan heptasaccharide, which would otherwise be detrimental to yields. Interestingly, in control samples lacking yeast, some loss of both galactose and XXXG was observed after extended incubation (96 h, Fig. 2B). This suggests possible contamination by other microorganisms from the laboratory environment, or from the TKP itself, which were resilient to the heating step.

Heat inactivation of the glycosidases conveniently allowed us to monitor the amount of XXXG during fermentation without more being produced. However, this heating step also constitutes an additional process complication. Thus, a comparable experiment was performed without enzyme inactivation (“direct fermentation”, Fig. 2C). HPLC analysis showed that the amount of galactose was reduced by over 90% in samples to which yeast were added, but this required an additional 18 h of fermentation to achieve (48 h, Fig. 2C *cf.* 30 h, Fig. 2B). Interestingly, the amount of XXXG increased by 20% over the entire course of this experiment (72 h), regardless of yeast addition. This suggested that xyloglucan was not completely hydrolyzed before the addition of yeast, and that retaining enzyme activity caused more XXXG to be produced. This also rationalized the additional time needed to ferment galactose, as the ongoing glycosidase-catalyzed hydrolysis would continue to generate more of the monosaccharide. It is worth noting that galactose was also removed partially in control samples to which yeast was not added. However, the amount removed was highly inconsistent (see error bars on grey squares, Fig. 2C), again suggesting the presence of other microorganisms in the crude TKP, which could utilize galactose (see “Prevention of unwanted fermentation products” section, below).

The fermentation experiments thus far were performed at room temperature (21 ºC, Fig. 2B and C). If desired, this step could be accelerated by using the optimal growth temperature of *S. cerevisiae* (30 ºC [62]), at the expense of requiring additional heating and temperature control. As a proof-of-principle to improve space–time yield, we performed the fermentation at this higher temperature. HPLC analysis showed that the amount of galactose was reduced below 5% after only 8 h at 30 ºC, versus 72 h at 21 ºC (Fig. 2D). The same increase in XXXG observed previously (Fig. 2C) also occurred within this shorter time-frame, with an even greater increase (*ca.* 35%) when extended to 24 h (Fig. 2D). Complete removal of galactose was also observed at this 24 h time-point. Interestingly, galactose was also removed in control digests to which yeast was not added, although the rate of galactose utilization was markedly slower. In general, although crude TKP may contain environmental microorganisms that can remove galactose from TKP digests, we anticipate that this would vary batch-to-batch, depending on industrial processing and storage conditions. Therefore, the routine addition of baker’s yeast is recommended to produce a more controlled process.

### Optimization of recombinant enzyme production

Based on our successful optimization of XXXG production from TKP by combined glycosidase digestion and fermentation at small scale (≤ 2.5 g), we were motivated to explore the possibility of scaling-up significantly (e.g., starting with 100 g of TKP). A foreseeable bottleneck was the production of sufficient quantities of recombinant *Bo*GH5A and *Cj*Bgl35A, which nonetheless we routinely produced at reasonable levels in shake flasks (*ca.* 20 mg of purified protein per liter of culture), using standard expression constructs in *E. coli* [51, 52]. In the first instance, we explored production via batch culture in a 2 L bioreactor, using identical parameters as in shake flasks, but controlling the dissolved oxygen level at 40%. Gratifyingly, the production of *Cj*Bgl35A with these conditions increased to 400 mg/L after purification. However, production levels of *Bo*GH5A did not increase.

We previously showed that full-length *Bo*GH5A contains two domains: An N-terminal spacer domain (*ca.* 10 kDa, Pfam PF13004) and a C-terminal GH5_4 catalytic domain (*ca.* 42 kDa) [51]. To improve production, we generated a new vector to produce only the catalytic domain (*Bo*GH5A_(cat)_) and switched the expression strain from *E. coli* BL21 DE3 to Rosetta DE3. This combination resulted in the production of *ca.* 100 mg of purified *Bo*GH5A from a 250 mL shake flask culture (i.e., 400 mg protein per liter of culture). Also gratifying, kinetic analysis showed that *Bo*GH5A_(cat)_ had similar specific activity on xyloglucan as the full-length enzyme (404 ± 10 µmol min^­1^ µmol *Bo*GH5A_(cat)_ versus 338 ± 7 µmol min^­1^ µmol *Bo*GH5A, 1 mg/mL pure tamarind xyloglucan, 21 ºC; *n* = 4) and produced the same product distribution with TKP (Figure S7).

### Prevention of unwanted fermentation products

**Fig. 3:**
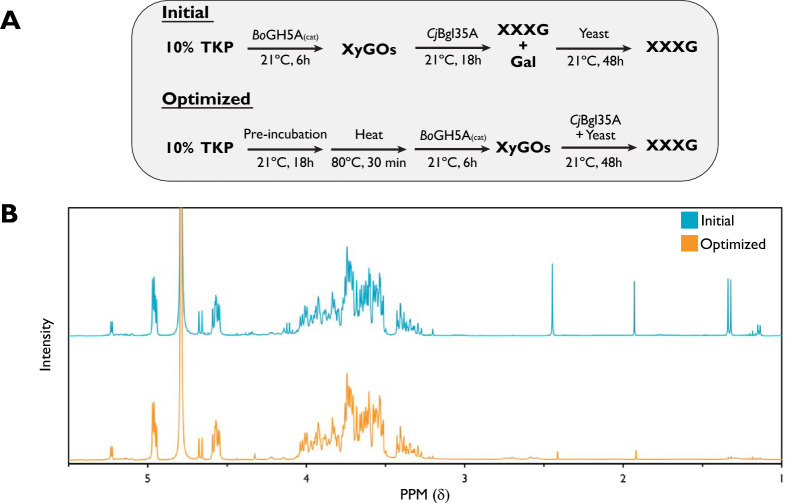
Optimization of large-scale enzymatic digestion of tamarind kernel powder (TKP). **A** Initial (serial addition of *Cj*Bgl35A β-galactosidase and yeast) and optimized (simultaneous addition) digestion schemes. **B** Proton NMR spectra of the final products obtained from the initial and optimized large-scale digests. *Bo*GH5A_(cat)_ was loaded at 0.2 mg/g TKP and *Cj*Bgl35A at 0.4 mg/g TKP

Following the optimization of galactose removal and recombinant enzyme production, we were keen to explore the potential to significantly scale-up the production of XXXG. Hence, 100 g of TKP in a 10% slurry was treated at room temperature with *Bo*GH5A_(cat)_ for 6 h followed by an overnight incubation with *Cj*Bgl35A (Fig. 3A). Yeast was then added to the digest and HPLC analysis was used to monitor the removal of galactose. Fermentation was allowed to proceed for 48 h, at which point no galactose was observed (data not shown, results similar to Fig. 2C). Following centrifugation to remove TKP residues and yeast cells, the supernatant was collected and lyophilized, yielding 44.6 g of XXXG. This mass was similar to the expected maximum yield based on the abundance of xyloglucan in TKP (50–60%) and accounting for the loss of mass due to galactose removal. This yield was also consistent with preliminary, smaller-scale (2.5 g) production runs (45.9 ± 3.5%, *n* = 2), indicating no significant losses upon scale-up.

The resulting XXXG was analyzed by ^1^H-NMR to provide an additional assessment of purity (Fig. 3B). The NMR spectrum was essentially identical to that reported for XXXG previously [46]. Surprisingly, however, significant signals were also observed at δ 1.10 (doublet), δ 1.29 (doublet), δ 1.88 (singlet), and δ 2.38 (singlet), corresponding to fermentation products 2,3-butanediol, lactic acid, acetic acid, and succinic acid, respectively [63]. Retrospective ^1^H-NMR analysis of a sample from the previous optimization experiments, to which baker’s yeast had not been added (Fig. 2D, 24 h sample), also revealed these same low-field signals (Figure S8). These data suggested the presence of one or more microorganisms contained within the bulk TKP preparation, which were capable of utilizing galactose through a mixed acid fermentation pathway that is not typically associated with baker’s yeast [64]. Indeed, culturing a sample of 10% TKP on yeast-peptone-galactose agar revealed growth of unidentified microorganisms with diverse colony morphologies (Figure S9). Although these non-volatile byproducts may not be an issue for some applications, preventing their formation is necessary to directly produce high-purity XXXG.

Consequently, we attempted to sterilize the TKP directly prior to enzyme addition using heat, including heating the solution of 10% TKP to 80 ºC for 30 min, boiling the solution using a microwave oven, and autoclaving the dry TKP prior to the addition of adding water. Drop assays on yeast-peptone-galactose agar performed after *Bo*GH5A_(cat)_ digestion showed no significant difference in microbial growth compared to untreated TKP (Figure S10A). ^1^H-NMR spectra of the final products after *Cj*Bgl35 addition and yeast fermentation also still contained signals corresponding to the unwanted fermentation products in 2.5 g test experiments (data not shown). These results suggested the presence of resistant microbial spores in the TKP that were difficult to eradicate.

A potential method to eradicate recalcitrant microbes involves inducing a vegetative state before treatment, which is sometimes referred to as the “germinate to eradicate” strategy [65]. To test this approach, a solution of 10% TKP was incubated at room temperature overnight, followed by heating to 80 ºC for 30 min. After enzyme treatment and yeast fermentation as before, comparative drop assays indicated a significant amount of growth in control samples (heating step omitted), which was almost completely eliminated after heat treatment (Figure S10B). Despite this reduction of colony-forming units, ^1^H-NMR analysis of 2.5 g test preparations unfortunately revealed significant continued production of the undesired fermentation products, although the distribution of these products was altered following the “germinate to eradicate” procedure (Figure S11A).

Given that this approach was only partially successful, we reasoned that additional microbes persist, and only germinate once galactose is released by *Cj*Bgl35. Therefore, we altered the procedure to introduce baker’s yeast immediately after the β-galactosidase. Gratifyingly, this approach effectively eliminated the production of 2,3-butanediol and lactic acid, and greatly reduced the amount of acetic acid in the sample (Figure S11B). This result suggested that baker’s yeast was able to effectively out-compete contaminating microbes in the TKP, metabolizing the liberated galactose to the desired volatile products, carbon dioxide and ethanol.

### Optimized procedure for XXXG production scale-up

Our combined analyses enabled us to devise an optimized procedure for XXXG production. Key steps included (1) production of a 10% slurry of TKP in water; (2) pre-incubation for 18 h at room temperature to induce germination of endogenous microbes; (3) brief heat treatment to reduce microbial load; (4) xyloglucan backbone hydrolysis; and (5) simultaneous degalactosylation and galactose fermentation (Fig. 3A). Initial application of this procedure on 2.5 g scale resulted in high yields of XXXG (52.0 ± 0.3%, *n* = 2) with an apparent purity comparable to that observed in initial optimization experiments (Figure S10B). Subsequent scale-up to 100 g TKP in 1 L of water, a level still practical in a laboratory setting, again allowed us to produce highly pure XXXG in 52.3% total yield (Fig. 3C). At this scale, we observed a slight turbidity of the sample after centrifugation to remove yeast cell and residual TKP solids, which could be removed by addition of activated carbon and filtering through celite (Figure S12).

## Conclusion

This study resulted in a facile, scalable method to produce Glc_4_-based XyGOs, and particularly the single Xyl_3_Glc_4_ heptasaccharide XXXG, from the bulk agricultural product TKP. The approach described here embodies goals of green chemistry by reducing solvent, material, and energy inputs consumption by applying enzyme- and cell-based biotransformations and avoiding the use of chromatography and organic solvents. A particular challenge was to overcome complications due to endogenous microbes in the TKP itself. The extent to which these may vary depending on industrial supplier and storage conditions is uncertain, but we anticipate that application of bulk sterilization techniques, such as ionizing radiation widely used in the food and medical industries, might be advantageous. If successful, this would further reduce energy inputs by removing the requirement for heating in the “germinate to eradicate” approach. Regardless, this research details an improved method to produce large quantities of XyGOs using normal laboratory-scale equipment, and also provides an outline for further scale-up. We anticipate that facile access to 100 g to kilogram quantities of highly pure XyGOs and XXXG can facilitate numerous bulk applications of these oligosaccharides.

## Materials and methods

### Materials

Ultrapure water with a resistivity of ρ > 18.2  MΩ cm (Millipore MilliQ system) was used for all experiments. De-oiled tamarind kernel powder (DTKP-60) was obtained from Premcem Gums, Private Limited (Mumbai, India; https://www.premcemgums.com/portfolio-item/tamarind-kernel-powder/). Cellulase from *Trichoderma reesei*, and β-galactosidase from *Aspergillus oryzae* were purchased as lyophilized powders from Sigma (product codes C8546, and G5160, respectively). β-galactosidase from *Aspergillus niger* was purchased as a suspension in 3.2 M ammonium sulfate from Megazyme (product code E-BGLAN). Commercial enzymes were added by protein mass (by weight for powders or concentration for suspensions), based on specific activity values provided by the suppliers. The production of recombinant enzymes is described in the following section. Fleischmann’s Original Active Dry Yeast (ACH Food Companies Inc., Tennessee, USA), purchased from a local supermarket was used as a source of *Saccharomyces cerevisiae*.

### Gene expression and protein purification

Full-length *Bo*GH5A was produced from previously transformed *E. coli* BL21 (DE3) cells [51]. DNA encoding the catalytic domain of *Bo*GH5A was amplified from the previous vector using the following primer pair: F-BoGH5A_(cat)_-LIC-AR TACTTCCAATCCAATGCATATAGCGTACCTGCATACG R-BoGH5A_(cat)_-LIC-AR TTATCCACTTCCAATGTTATTAATTTAATCCTTTCATAATCGCTGC. After cloning into pMCSG53, the plasmid was transformed into *E. coli* Rosetta (DE3) cells following the manufacturer’s protocol (Millipore Sigma). DNA encoding *Cj*Bgl35A was re-cloned from pET28a [52] into pMCSG53 and transformed into *E. coli* Rosetta (DE3) cells following the manufacturer’s protocol (Millipore Sigma). Whole plasmid sequencing was performed by Plasmidsaurus Inc. (https://www.plasmidsaurus.com/) using Oxford Nanopore Technology with custom analysis and annotation.

Overnight cultures for each production clone were grown at 37 ºC and 200 RPM in TB liquid media (24 g/L yeast extract, 12 g/L tryptone, 0.4% glycerol). The next day, a baffled shake flask with fresh TB was inoculated using 1:1000 the volume of overnight culture. The optical density at 600 nm (OD600) was monitored using a cell density photometer (Cole-Palmer, Canada). Protein expression was induced by adding isopropyl β-D-1-thiogalactopyranoside (IPTG) to a final concentration of 0.5 mM once an OD600 between 0.5 and 0.8 was reached. The cultures were left to grow at 25 ºC for a further 24 h to express *Cj*Bgl35A and 48 h to express *Bo*GH5A. *Bo*GH5A_(cat)_ was expressed at 16 ºC for 48 h. The same conditions were used for expression from a 3 L Applikon Bioreactor attached to a Bio Controller (ADI 1010), with dissolved oxygen being controlled at 40%. Cell pellets were collected by centrifugation at 2500 g and frozen at – 70 ºC.

Pellets were thawed and resuspended in buffer A (20 mM sodium phosphate pH 7.4, 500 mM NaCl, 20 mM imidazole) then sonicated to disrupt cellular membranes. The supernatant containing soluble protein was separated by centrifugation at 4 ºC (24,000 g for 1 h), and passed through a 0.45 µm PVDF membrane (Millex^®^-HV). The recombinant enzymes, which contained a 6X Histidine tag, were purified from the lysate using a HisTrap HP column (GE Healthcare) on a BioRad fast protein liquid chromatography system with an elution gradient of 0–100% buffer B (20 mM sodium phosphate pH 7.4, 500 mM NaCl, 500 mM imidazole). The purified proteins were concentrated and exchanged into 50 mM sodium phosphate buffer (pH 6.0) using a Vivaspin^®^ 20 10 kDa molecular weight cut-off spin concentrator. The purity of each recombinant enzyme was confirmed by SDS-PAGE. The concentration of recombinant proteins were determined from their calculated molar extinction coefficients (ProtParam at ExPASy [66]) at 280 nm using an Epoch microplate spectrophotometer (BioTek). Recombinant proteins were flash frozen in 100 µL aliquots using liquid nitrogen and stored at – 70 ºC until use.

### Determination of recombinant endo-xyloglucanase activity

To determine any catalytic effect due to truncation of the full-length enzyme [51], the specific activities of *Bo*GH5A and *Bo*GH5A_(cat)_ *endo*-xyloglucanases were quantified using a bicinchoninic acid (BCA) reducing sugar assay [67]. Each enzyme was used at 0.5 µg/mL. The reactions were conducted in quadruplet in a final volume of 100 µl in 50 mM sodium phosphate buffer (pH 6.0) at 21 ºC for 10 min with 1 mg/mL tamarind xyloglucan (Megazyme, Lot#100403). Reactions were terminated with the addition of an equal volume of BCA working reagent. Color was developed at 80 ºC for 20 min, and the absorbance was read at 563 nm. A series of glucose concentrations (5 to 100 µM) was included in the assay to quantify the amount of reducing ends released. Blank reactions containing only substrate in assay buffer were used to measure the background absorbance generated by reducing ends present on the undigested tamarind xyloglucan. The enzyme concentration used in this assay was not high enough to contribute significantly to the absorbance at 563 nm.

### Carbohydrate analysis

Carbohydrate quantification was preformed using High Performance Anion-Exchange Chromatography with Pulsed Amperometric Detection (HPAEC-PAD) using a Dionex ICS-6000 DC HPLC with a Dionex PA200 Carbopac guard and analytical column in series. Samples were prepared by diluting in water followed by centrifugation through a costar^®^ SpinX 0.2 µm cellulose acetate filter column, then transferred to an HPLC vial. 10.0 µL of sample was injected by an AS-AP autosampler (Dionex).

Elution solvent A was ASTM filtered water, solvent B was 1 M sodium hydroxide, and solvent D was 1 M sodium acetate. Flow rate was 0.5 mL/min. Gradient A: 0 min (10% B, 6% D, curve = 5), 4 min (10% B, 6% D, curve = 5), 17 min (10% B, 35% D, curve = 5), 17.1 min (50% B, 50% D, curve = 5), 18 min (10% B, 6% D, curve = 9), then 22 min (10% B, 6% D, curve = 5). Gradient B: 0 min (10% B, 3.5% D, curve = 5), 4 min (10% B, 3.5% D, curve = 5), 12 min (10% B, 30% D, curve = 5), 12.1 min (50% B, 50% D, curve = 5), 13 min (10% B, 3.5% D, curve = 9), then 22 min (10% B, 3.5% D, curve = 5). Data analysis was performed with Chromeleon software (Dionex) to integrate peak areas. Peaks for galactose and XyGOs were assigned based on elution times of standards [5, 45].

Mass spectrometry was performed by using a Bruker Autoflex matrix-assisted laser desorption/ionization time of flight mass (MALDI-TOF) spectrometer. Samples were dissolved in water to 0.1 mg/mL before mixing with an equal volume of 2,5-dihydroxybenzoic acid dissolved to 20 mg/mL in acetonitrile.

Proton nuclear magnetic resonance (^1^H-NMR) was preformed using a Bruker Avance 400inv spectrometer. Samples were dissolved to 10 mg/mL in deuterium oxide for analysis.

### Initial enzyme specificity screening

A 250 mL round bottom flask was filled with 125 mL of water to which 5 g of TKP was added portion-wise while stirring at 60 ºC. The mixture was cooled to room temperature and either 0.84 mg of *Bo*GH5A or 25 mg (6 U/mg; one unit is defined by the supplier as liberating 1.0 µmole of glucose from cellulose in 1 h at pH 5.0 at 37 °C) of cellulase from *T. reesei* was added (loadings determined empirically, based on test reactions and previous experience [32, 51]). The reactions were allowed to proceed for 24 h before being heated to 80 ºC for 15 min to inactivate enzymes. The enzyme digestion was repeated with *Bo*GH5A with no heat inactivation step, 1 mg of either *Cj*Bgl35A, *A. oryzae*, or *A niger* β-galactosidase was subsequently added, and the reaction was allowed to continue for 24 h before heat inactivation. Samples were centrifuged to remove insoluble particulate, and a sample of the supernatant was diluted 1:100 for analysis with HPAEC-PAD using Gradient A. Following lyophilization (Labconco FreeZone 4.5 L–105 °C benchtop freeze dryer), the sample was analyzed by MALDI-TOF MS analysis.

### Optimizing TKP concentration

TKP was added to 25 mL of water to a final concentration of 4, 10, 15, or 20% (w/v) to which 0.2 mg/g TKP of *Bo*GH5A was added. Samples were left at room temperature (21–22 °C) on a Corning LSE^™^ platform rocker at 100 rocks per minute. After 24 h, 0.4 mg/g TKP of *Cj*Bgl35A was added to each sample and the reaction was allowed to continue for another 24 h. Samples were then centrifuged at 1300 g for 15 min. The supernatant was transferred to pre-weighed tubes and heated to 80 ºC for 20 min to inactivate the enzymes. The samples were then frozen to − 70ºC and lyophilized. Final products were weighed to calculate yield and analyzed using HPAEC-PAD (Gradient B) to ensure that complete digestion into XXXG and galactose was reached.

### Time-course analysis of enzyme hydrolysis

A 25 mL 10% w/v TKP solution was prepared. 0.5 mg of *Bo*GH5A was added to the solution then it was left at room temperature (21–22 °C) on a Corning LSE^™^ platform rocker at 100 rocks per minute. 200 µL samples were taken before, and every hour after addition of the enzyme. Samples were heated to 80 °C for 20 min to inactivate enzymes before centrifugation to collect supernatant. Supernatant was diluted 1:200 before HPAEC-PAD analysis (Gradient B). The same experiment was set up again. *Bo*GH5A digestion went for 6 h before the adding 1 mg of *Cj*Bgl35A. Samples were taken before, and every hour after, *Cj*Bgl35A addition.

### Purification by selective precipitation

Samples of TKP (2.5 g in 25 mL water) were treated with 0.5 mg *Bo*GH5A for 6 h followed by 1 mg of *Cj*Bgl35A for 18 h. Supernatants were collected by centrifugation at 1300 g for 15 min and divided into 500 µL aliquots, to which ethanol was added in ratios of 1:1, 1:5, 1:10, and 1:20. Samples were vortexed and centrifuged at 1300 g for 15 min. 100 µL of supernatant was collected from each sample for HPAEC-PAD analysis (Gradient B) and the rest was discarded. The pellets were washed once with 95% ethanol. All pellets and supernatants were then dried at 37 ºC for 18 h. The residues were resuspended in water (500 µL for pellet samples, 200 µL for supernatant samples). The pellet samples were then diluted 1:1000 and the supernatant samples were diluted 1:250, 1:100, 1:50, and 1:25 for the ethanol precipitation ratios 1:1, 1:5, 1:10, and 1:20, respectively, to keep samples at the same dilution factor. Peak areas from HPAEC-PAD chromatograms (Gradient B) were used to calculate the relative amount of XXXG and galactose in the supernatant and pellet for each sample.

### Galactose removal by yeast metabolism

The package of dry yeast was opened and the pellets were revived by adding them to YPG media (10 g/L yeast extract, 20 g/L Bacto^®^ peptone, 2% D-galactose) and growing at 30 ºC and 200 RPM for 8 h. The growing yeast was then sub-cultured into fresh YPG media and made into glycerol stocks during mid-log phase (optical density @ 600 nm = 0.4). Cells from the frozen stock were streaked onto YPG agar and grown at room temperature overnight. A single colony was used to inoculate fresh YPG media (20% of the TKP digestion volume) and left to grow overnight at room temperature. Cells were centrifuged at 1300 g and 4 ºC then washed once with an equal volume of PBS (137 mM NaCl, 2.7 mM KCl, 10 mM Na_2_HPO_4_, 1.8 mM KH_2_PO_4_, pH 7.4). The cell pellet was then resuspended with an aliquot of the ongoing TKP digestion reaction and added back into the reaction vessel. Fermentation of galactose was allowed to proceed at room temperature or 30 ºC. Galactose removal from samples was assessed using HPAEC-PAD (Gradient B) following centrifuging at 1300 g for 15 min, heating the supernatant to 80 °C for 20 min to inactivate enzyme activity, and diluting 1:500 for analysis.

### Microbial growth assays

Initial microbial content of tamarind kernel powder was assessed by plating a solution of 10% w/v tamarind kernel powder on yeast-peptone-galactose agar (1.5%) and growing at room temperature for 48 h. Images were taken using a smartphone camera.

Microbial drop assays were used to assess the microbial growth in tamarind kernel powder solutions across various treatments. Samples were taken from TKP digestions following a 6 h treatment by *Bo*GH5A_(cat)_ (0.2 mg/g TKP) and serially diluted using sterile water. 2.5 µL of each dilution was dropped onto yeast-peptone-galactose agar and then grown at room temperature (21–22 ºC) for 48 h. Plates were imaged on a Cell Biosciences’ red^®^ imaging system.

### Optimized XXXG preparation

A TKP solution (10% w/v) was prepared in sterile water. At the same time, a culture of yeast in YPG media (20% TKP solution volume) was inoculated using a single colony on YPG agar and grown at 25 °C and 150 RPM. The TKP solution was pre-incubated by stirring with a magnetic stir bar for 18 h at room temperature (21–22 °C). The solution was then heated to 80 °C for 30 min and subsequently cooled to room temperature. Recombinant *Bo*GH5A_(cat)_ was added to loading of 0.2 mg/g TKP. The solution was then stirred for 6 h before *Cj*Bgl35A was added to a loading of 0.4 mg/g TKP. Yeast cells from the initial culture were pelleted, washed, and added to the reaction immediately following the addition of *Cj*Bgl35A. Removal of galactose after 48 h was confirmed by HPAEC-PAD (Gradient B), at which point yeast and insoluble TKP residues were removed by centrifugation at 1300 g for 15 min. The supernatant was heated to 80 °C for 30 min to inactivate the enzymes and lyophilized to yield a dry powder.

### Supplementary Information

Supplementary Material 1

## Data Availability

All data supporting the conclusions of this article are included within the article and its additional files. Non-commercially available materials are available from the authors upon reasonable request. No datasets were generated or analysed during the current study.

## Abbreviations

XXXG: Xyloglucan heptasaccharide [α-D-Xyl*p*-(1 → 6)]-β-D-Glc*p*-(1 → 4)-[α-D-Xyl*p*-(1 → 6)]-β-D-Glc*p*-(1 → 4)-[α-D-Xyl*p*-(1 → 6)]-β-D-Glc*p*-(1 → 4)-D-Glc*p*).
XyG: Xyloglucan
XyGO: Xyloglucan oligosaccharide
*Bo*GH5A: *Bacteroides ovatus* glycoside hydrolase family 5 *endo*-xyloglucanase
*Cj*Bgl35A: *Cellvibrio japonicus* glycoside hydrolase family 35 β-galactosidase
TKP: Tamarind kernel powder
