# Supplementary material for: A scalable, chromatography-free, biocatalytic method to produce the xyloglucan heptasaccharide XXXG
Source: Biotechnol Biofuels Bioprod. 2024 Aug 20;17:116. doi: 10.1186/s13068-024-02563-9 (PMC11337882; doi:10.1186/s13068-024-02563-9)
Supplement: Supplementary file 1 — Supplementary Material 1 [file 13068_2024_2563_MOESM1_ESM.pdf]

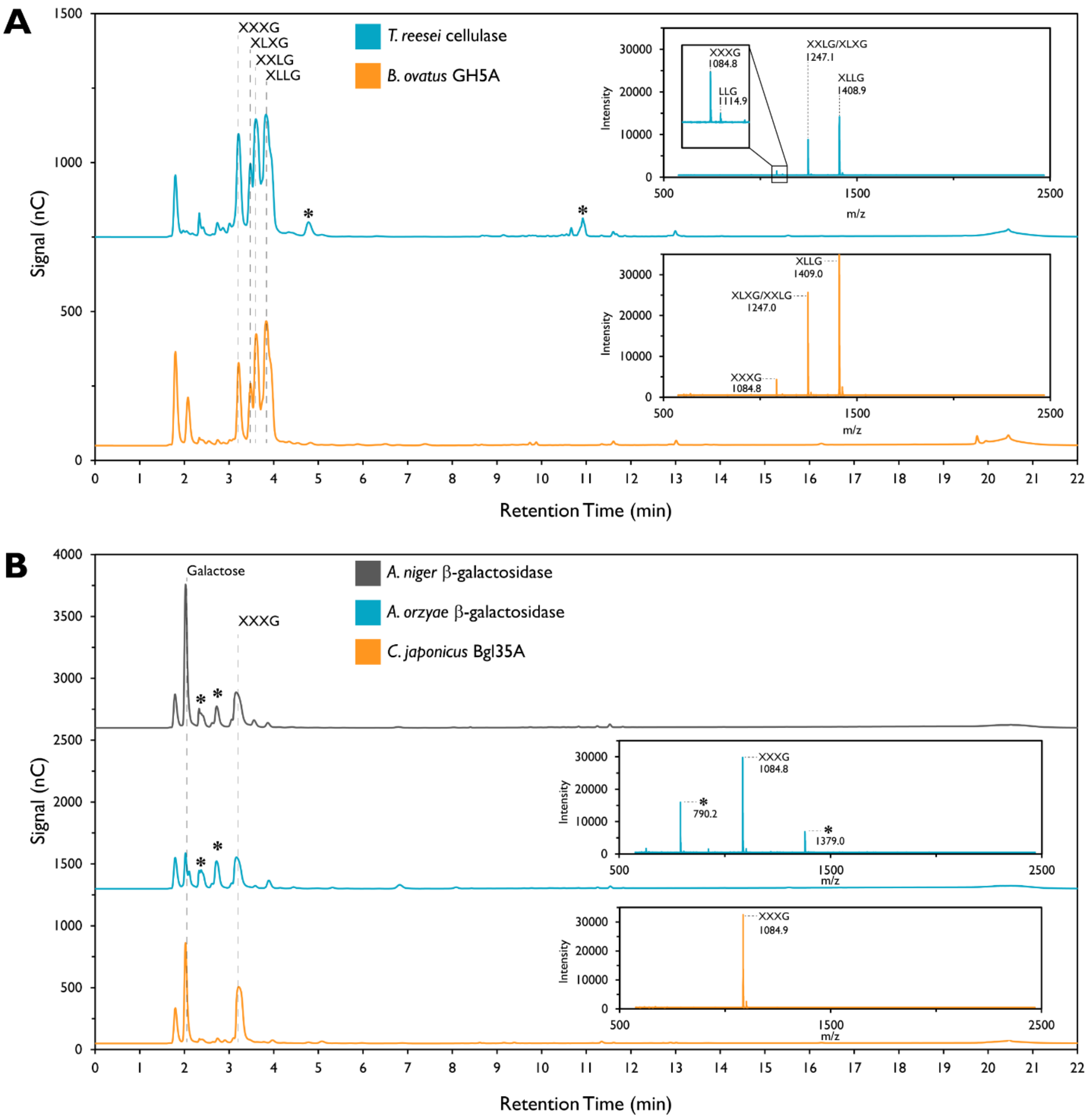

Figure S1: Products of commercial fungal and recombinant bacterial enzyme hydrolysis tamarind kernel powder xyloglucan. HPAEC-PAD chromatograms and MALDI-TOF mass spectra (insets) for **(A)** *endo*-(xylo)glucanase hydrolysis and **(B)** β-galactosidase hydrolysis. Signals from known saccharides are labelled specifically, asterisks indicate unidentified oligosaccharides.

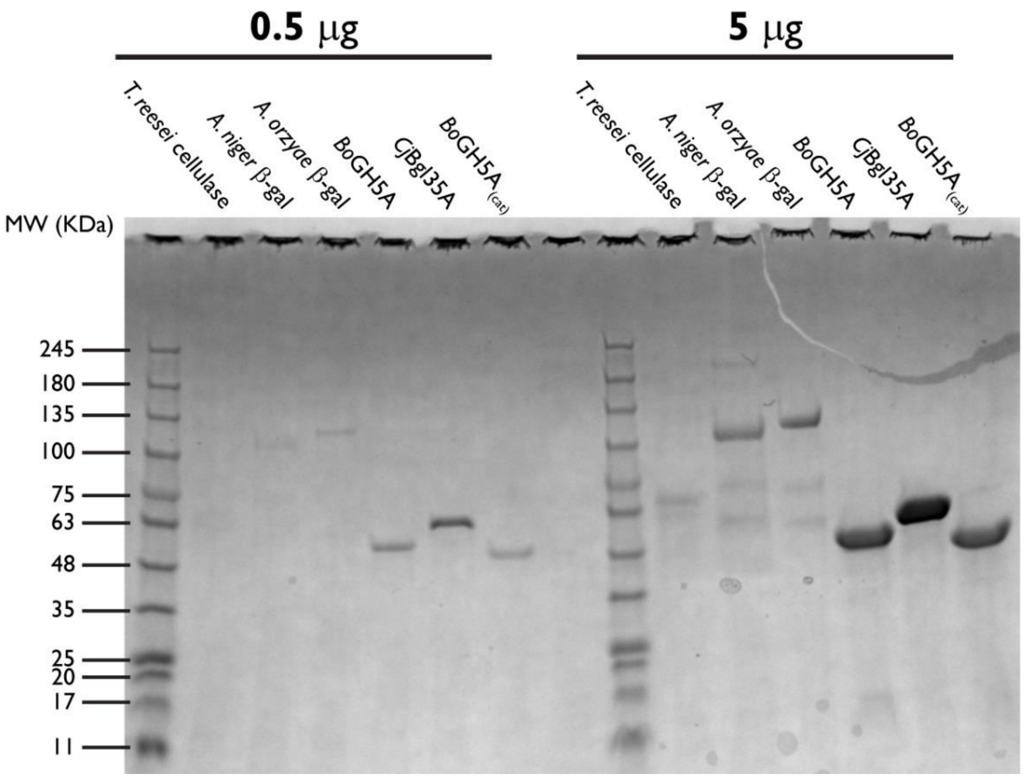

Figure S2: SDS-PAGE analysis of the enzyme preparations used in this study. Samples were loaded in equal amounts to a Bio-Rad Mini-PROTEAN®TGX™ 4-20% precast gel. The gel was stained using Coomassie Brilliant Blue R-250 (Bio-Rad) and imaged using a ChemiDoc XRS+ System (BioRad). Electrophoretic mobilities were compared with FroggaBio BLUelf Prestained Protein Ladder with molecular weight (MW) values indicated.

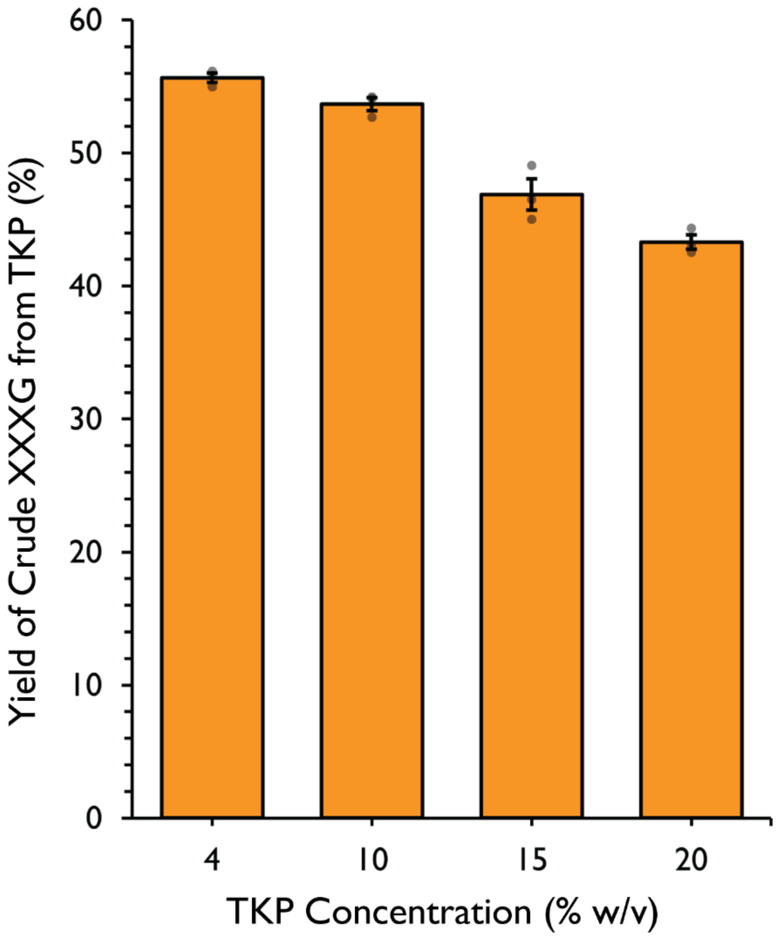

Figure S3: Yield of crude XXXG and galactose from the enzymatic digestion of tamarind kernel powder (TKP) at various slurry concentrations. TKP was digested for 24 h using *Bo*GH5A (0.2 mg/g TKP) followed by 24 h with *Cj*Bgl35A (0.4 mg/g TKP). The final mass of each sample was measured after lyophilizing the supernatant from each digest. Each bar represents the average of three independent assays, with error bars representing the standard error of the mean.

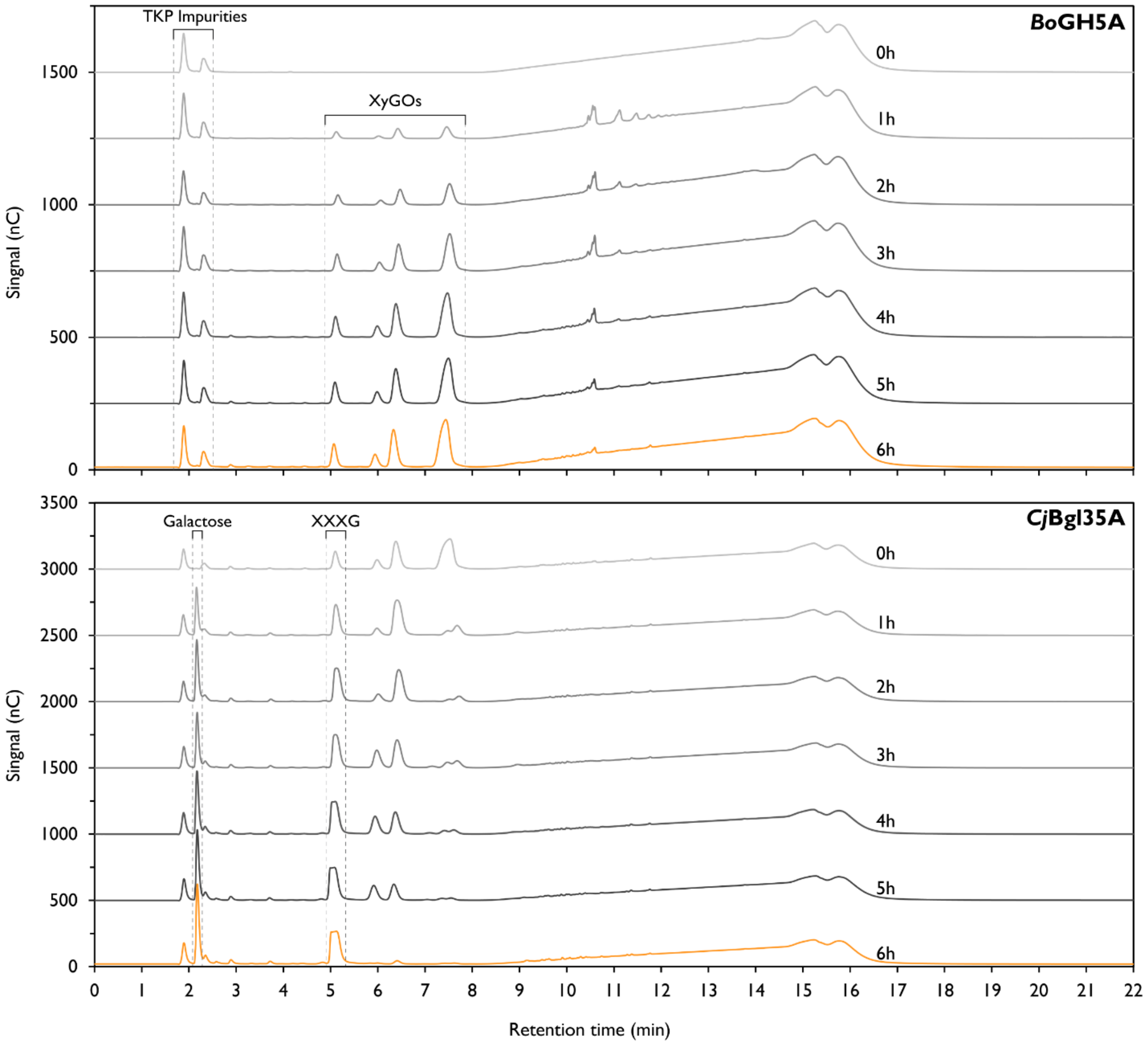

Figure S4: HPAEC-PAD analysis of tamarind kernel powder (TKP) xyloglucan hydrolysis using *endo*-xyloglucanase *Bo*GH5A (0.2 mg/g TKP) and β-galactosidase *Cj*Bgl35A (0.4 mg/g TKP). A 10% slurry of TKP was treated for 6 h at room temperature (21-22ºC) with each enzyme in series.

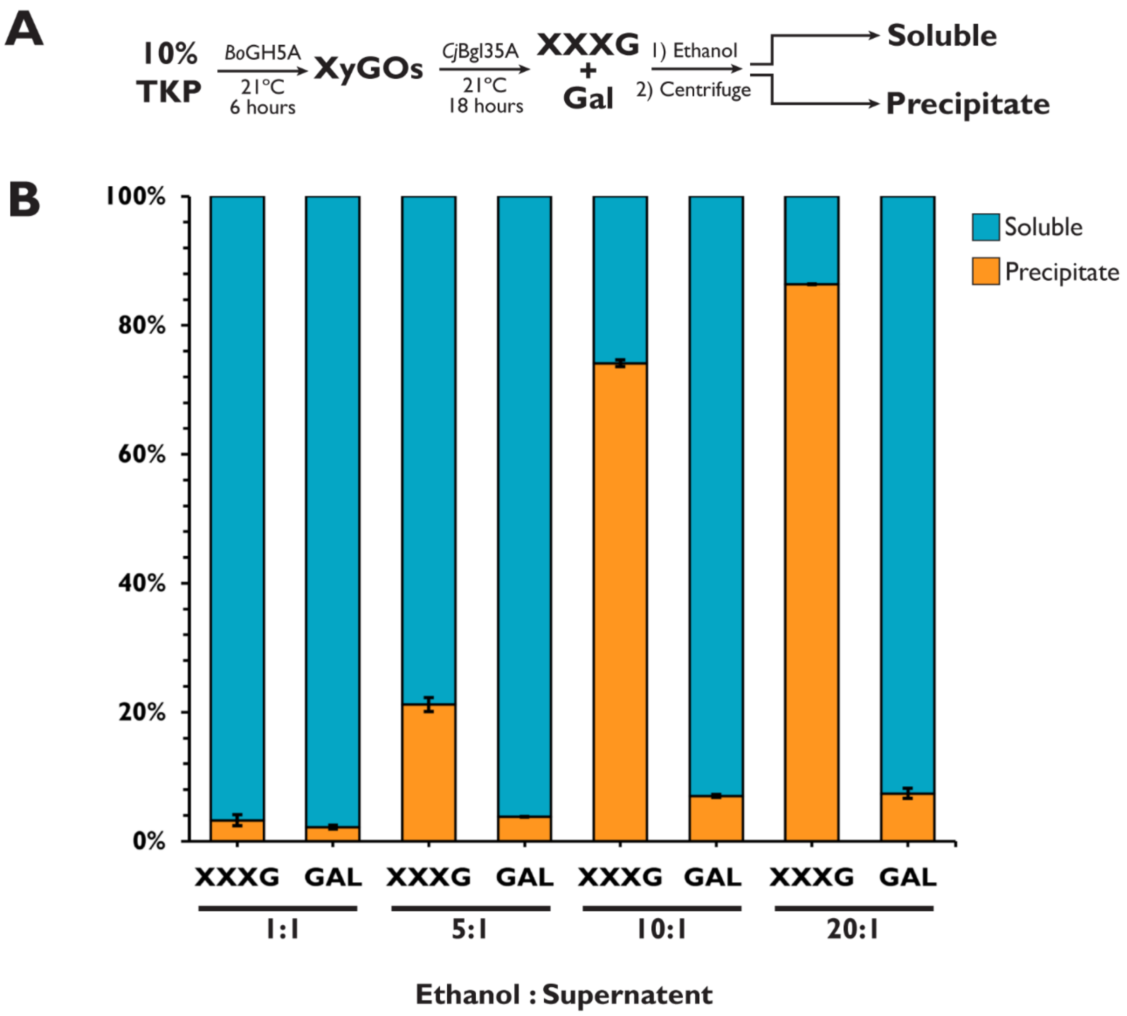

Figure S5: Removal of galactose from tamarind kernel powder (TKP) enzymatic digestions using selective precipitation with ethanol. (A) Process scheme. *Bo*GH5A was loaded at 0.2 mg/g TKP and *Cj*Bgl35A at 0.4 mg/g TKP. **(B)** Percentage of XXXG and galactose found in soluble and precipitated fractions following ethanol addition to supernatant at various ratios, quantified by HPAEC-PAD. Insoluble particulates were first removed by centrifugation prior to ethanol addition. Each bar represents the average from two independent experiments.

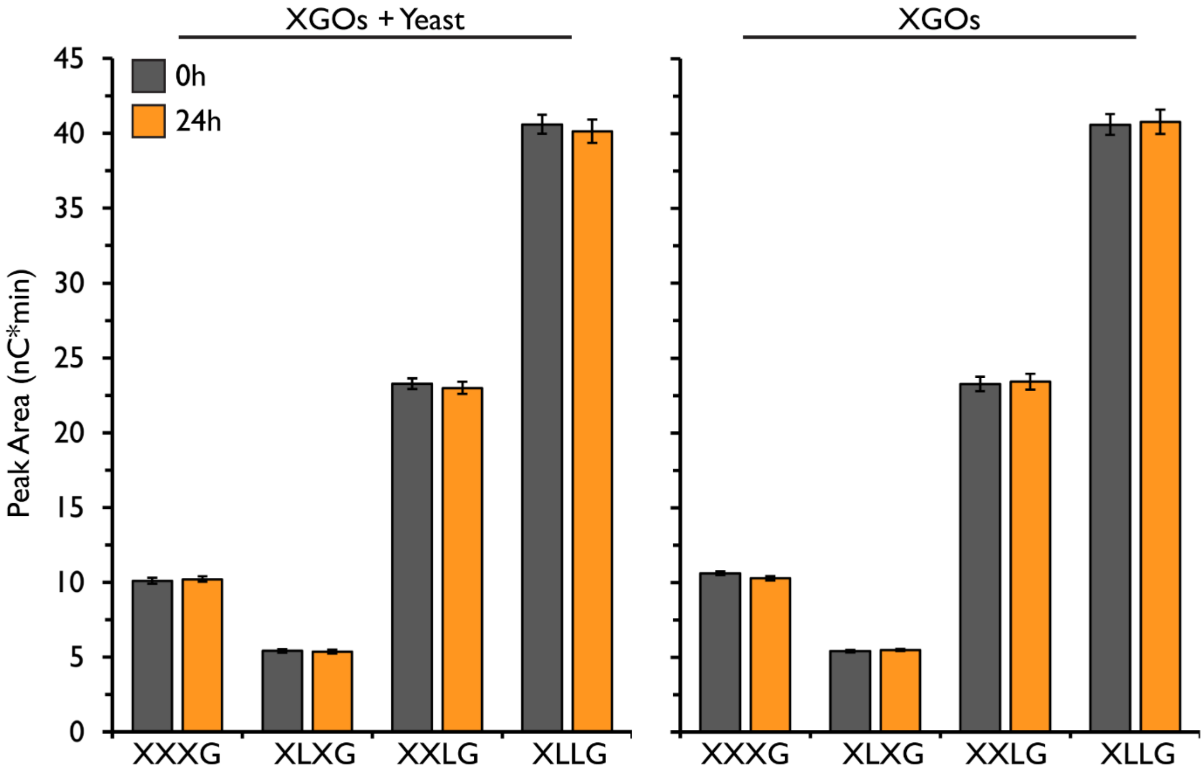

Figure S6: Lack of degradation of galactosylated xyloglucan oligosaccharides by *S. cerevisiae* used in this study. 10% TKP was hydrolyzed with *Bo*GH5A$_{(cat)}$ (0.2 mg/g TKP) for 18 h followed by centrifugation. The supernatant was collected and heated to 80ºC for 15 min. After cooling, the supernatant was incubated at room temperature (21-22ºC) for 24 h with (left) or without (right) addition of washed yeast cells. HPAEC-PAD was used to measure the relative amounts of XyGOs to monitor galactose removal. Each bar represents the average of three independent assays, with error bars representing the standard error of the mean.

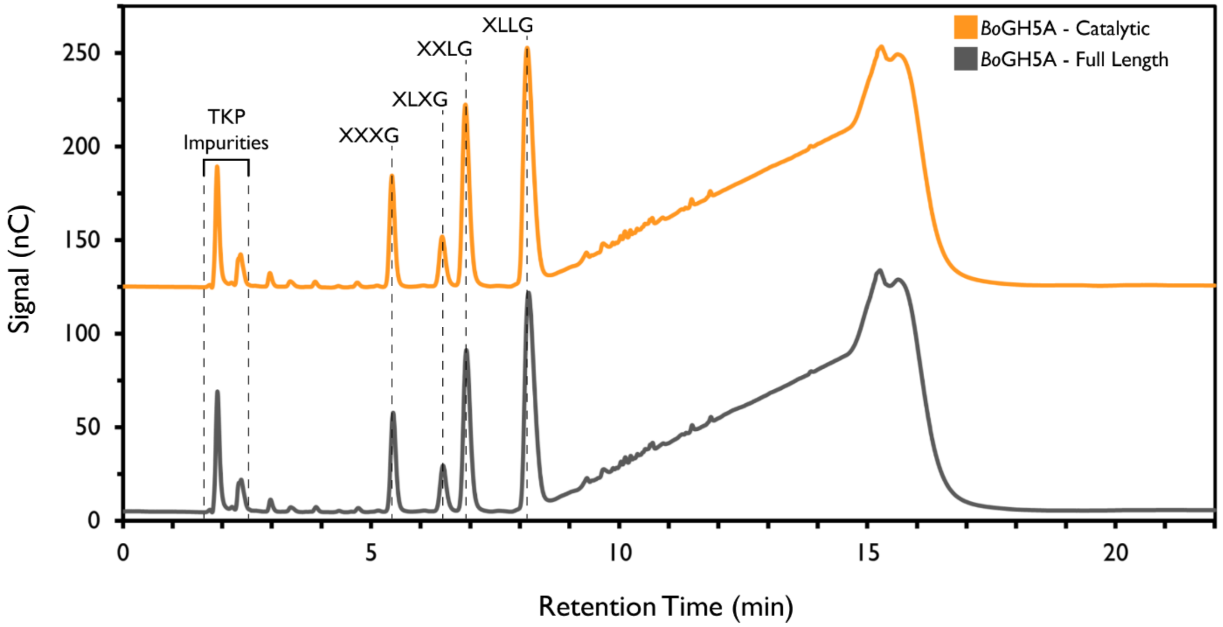

Figure S7: Digestion of tamarind kernel powder xyloglucan using full-length *Bo*GH5A or the isolated catalytic domain, *Bo*GH5A$_{\mathbf{(cat)}}$. The digestion was carried out for 24 h at room temperature (21-22ºC) with 0.2 mg enzyme/g TKP.

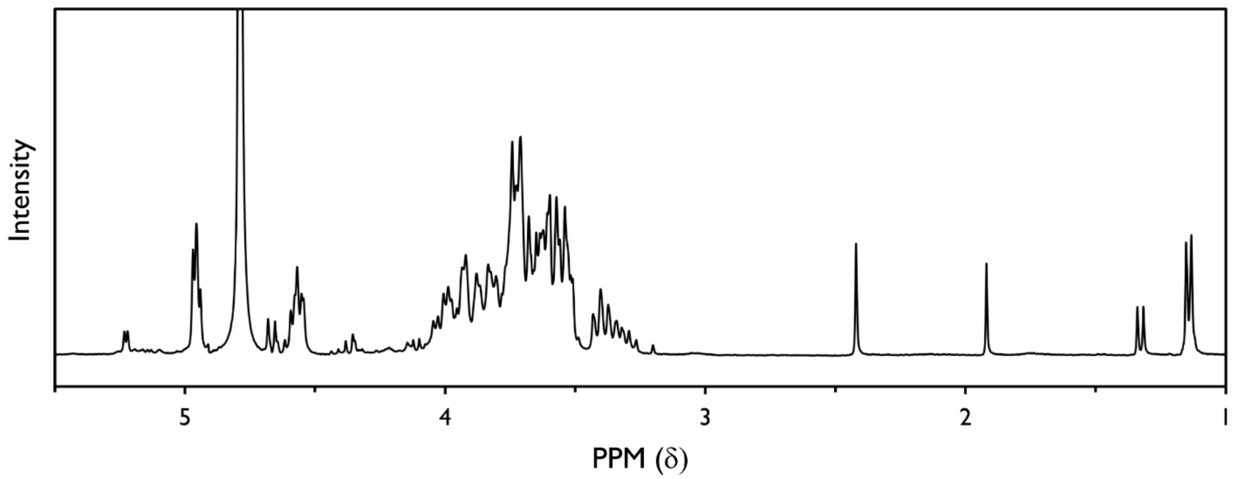

Figure S8: Proton NMR spectra of XXXG obtained from the enzymatic digestion of tamarind kernel powder without the addition of yeast. All three replicates from the 24 h time-point in Figure 2D were pooled together, lyophilized, and re-dissolved to 10 mg/mL in deuterium oxide. Signals at δ 1.10 (doublet), δ 1.29 (doublet), δ 1.88 (singlet), and δ 2.38 (singlet), correspond to 2,3-butanediol, lactic acid, acetic acid, and succinic acid, respectively, presumed to arise from fermentation of galactose by microbes in the commercial TKP preparation.

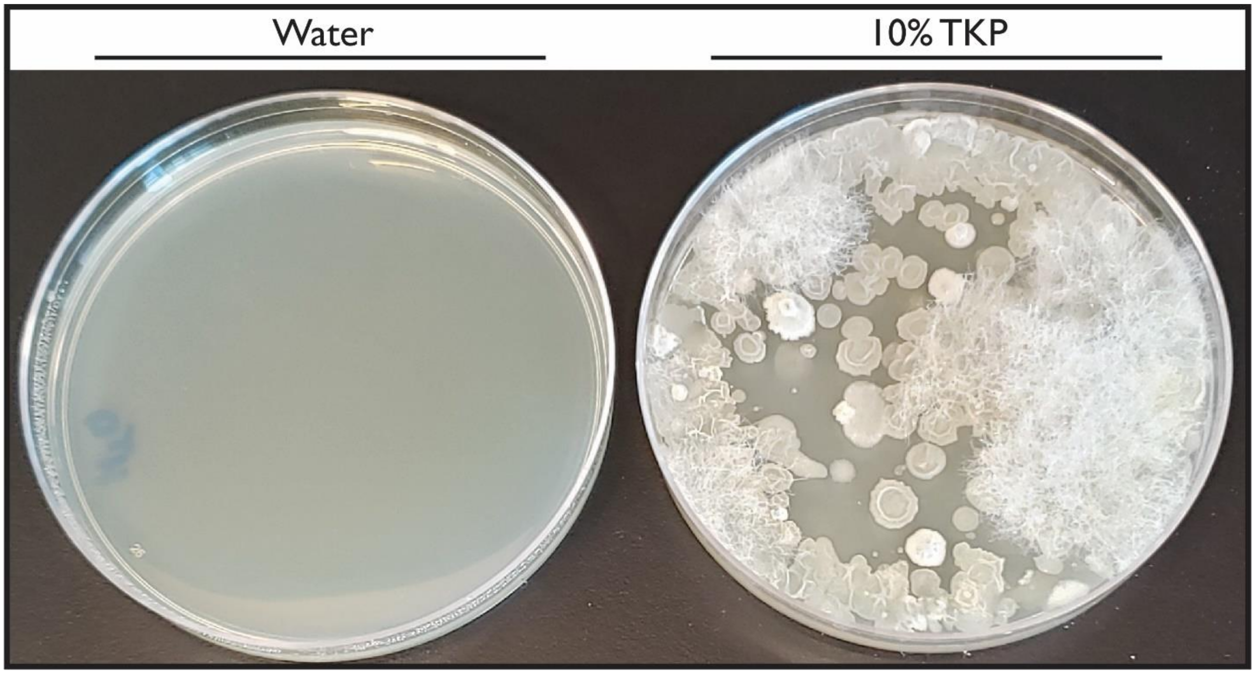

Figure S9: Microbes present in the commercial tamarind kernel powder (TKP) used in this study. A 200 µL aliquot of a 10% TKP solution was spread onto yeast-peptone-galactose agar and incubated at room-temperature (21-22ºC) for 48 h (right plate). 200 µL of the water used to prepare the solution was also spread onto a plate as a control (left plate).

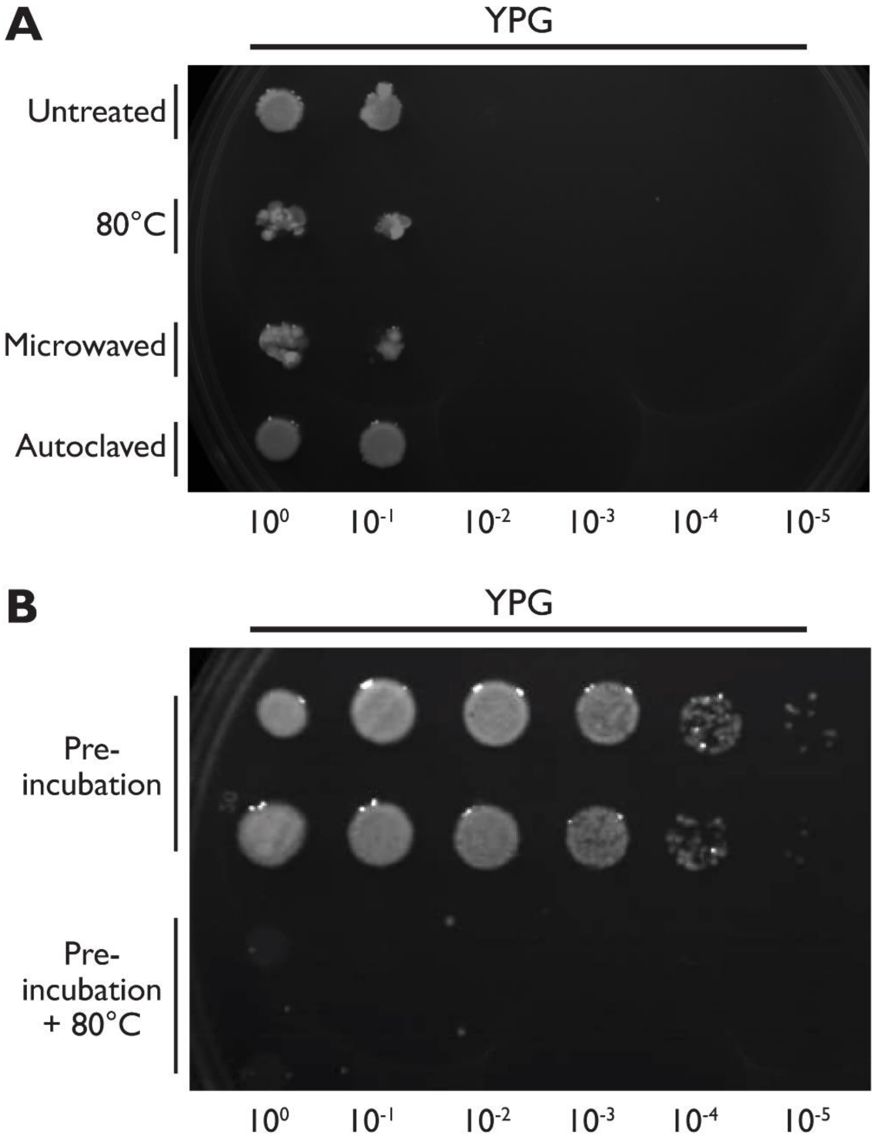

Figure S10: Semi-quantitative assays of microbial growth from tamarind kernel powder (TKP). 10% TKP slurries were treated and digested with 0.2 mg *Bo*GH5A$_{(cat)}$/g TKP before 10-fold serial dilution and spotting (2.5 µL) onto yeast-peptone-galactose (YPG) agar. Plates were incubated at room temperature (21-22ºC) for 48 h. **(A)** TKP slurry, TKP slurry heated to 80ºC for 30 mins, TKP slurry microwaved until boiling, and autoclaved dry TKP. **(B)** Pre-incubation of TKP slurry for 18 h at room-temperature and after heating to 80ºC for 30 min following the pre-incubation.

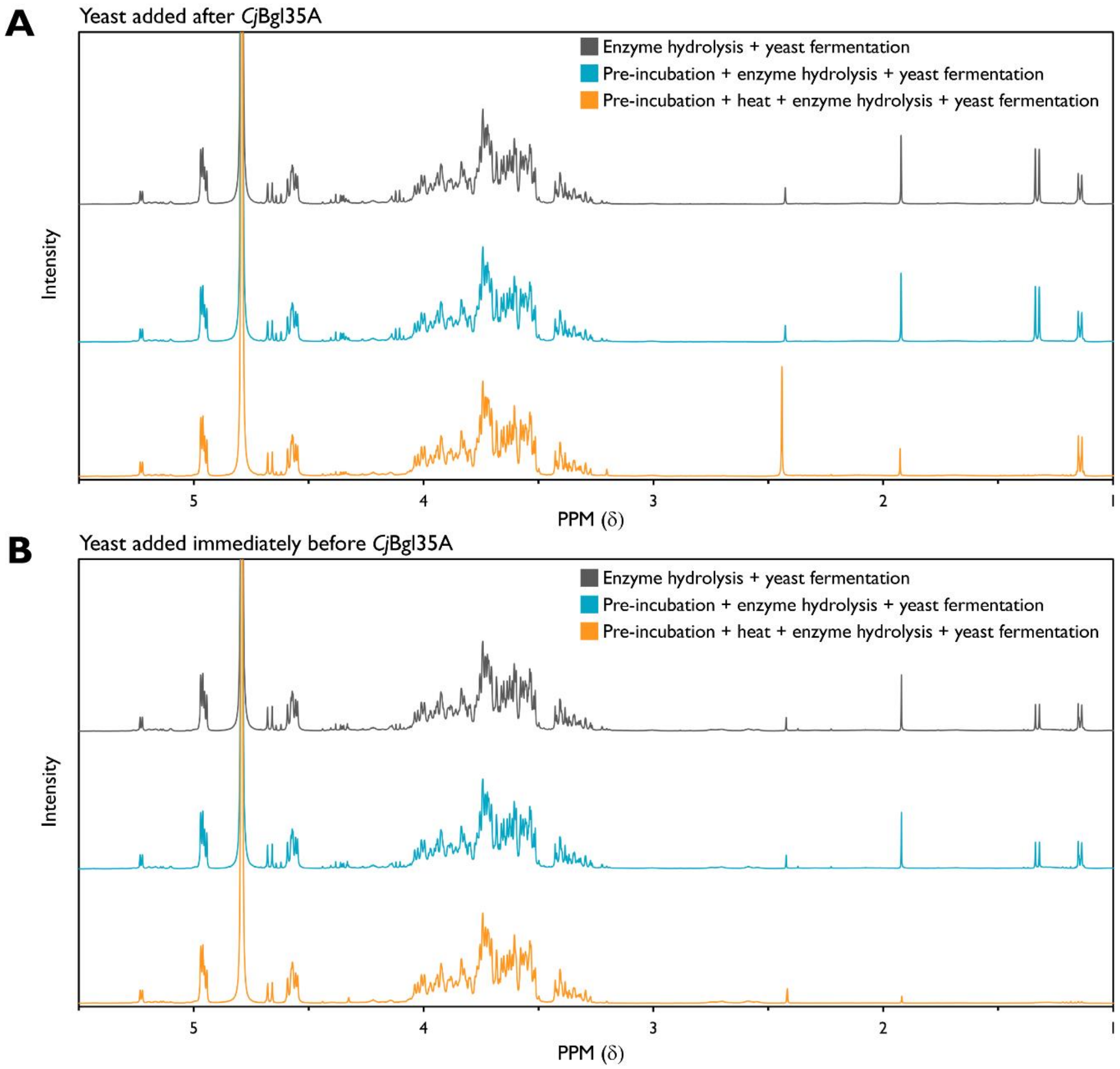

Figure S11: Assessment of fermentation biproduct production using ^{1}H-NMR in tamarind kernel powder digestions with pre-incubation and/or heat treatment. 10% TKP was pre-incubated for 18 h at room temperature (21-22ºC) followed by heating to 80ºC for 30 min. The solution was then hydrolyzed with *Bo*GH5A$_{(cat)}$ (0.2 mg/g TKP) for 6 h, followed *Cj*Bgl35A (0.4 mg/g TKP) for 18 h. Galactose was removed by fermentation with yeast added simultaneously **(A)** or after **(B)** CjBgl35A treatment. Samples were lyophilized and redissolved to 10 mg/mL in deuterium oxide.

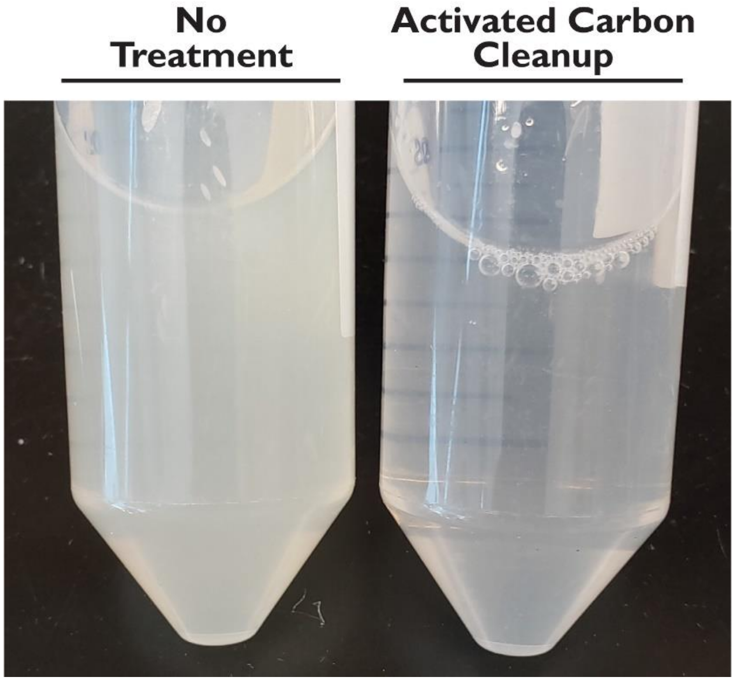

Figure S12: Clarification of XXXG solutions by activated carbon treatment and filtration. The left tube contains a sample of XXXG from a 100 g-scale digestion of TKP, immediately following centrifugation to remove insoluble TKP residues and yeast cells. The right tube shows an analogous 25 mL sample which was treated with 0.5 g of activated carbon (0.2 g activated carbon/g TKP) for 15 min and vacuum filtered through celite.
